# A Case of Pleomorphic Adenoma of the Submandibular Gland Radiologically Suspected to be Malignant

**DOI:** 10.7759/cureus.89009

**Published:** 2025-07-29

**Authors:** Noriyuki Sugino, Yutaka Kitamura, Katsumitsu Shimada, Hiroko Kuroiwa, Akira Taguchi

**Affiliations:** 1 Department of Oral and Maxillofacial Radiology, School of Dentistry, Matsumoto Dental University, Shiojiri, JPN; 2 Department of Oral and Maxillofacial Surgery and Dental Implant, Center of Oral and Maxillofacial Surgery and Dental Implant in Shinshu, Obuse, JPN; 3 Department of Clinical Pathophysiology, School of Dentistry, Matsumoto Dental University, Shiojiri, JPN; 4 Department of Pediatric Dentistry, School of Dentistry, Matsumoto Dental University, Shiojiri, JPN

**Keywords:** capsule, differential diagnosis, pleomorphic adenoma, salivary gland tumor, submandibular gland

## Abstract

Pleomorphic adenoma (PA) is the most common benign salivary gland tumor, typically arising from the parotid gland. PA of the submandibular gland is relatively uncommon and may present diagnostic challenges, particularly when imaging findings raise suspicion of malignancy. A 66-year-old woman presented with a painless mass in the left submandibular region. Imaging studies including unenhanced computed tomography (CT), magnetic resonance imaging (MRI), ultrasonography (US), and positron emission tomography (PET) revealed a lobulated mass with irregular margins, heterogeneous internal architecture, and partially disrupted capsular structures. PET showed abnormal fluorodeoxyglucose (FDG) accumulation with a maximum standardized uptake value (SUVmax) of 3.70. Based on these findings, malignancies such as adenoid cystic carcinoma or carcinoma ex pleomorphic adenoma (CXPA) were strongly suspected. The tumor was resected under general anesthesia with careful preservation of the capsule and excised en bloc together with the submandibular gland and a portion of the sublingual gland. Histopathological and immunohistochemical analyses revealed no evidence of malignancy, and a final diagnosis of PA was made. This case highlights the diagnostic difficulty of submandibular PA, especially when capsular structures appear ambiguous on imaging. It underscores the limitations of relying solely on imaging modalities and reaffirms the importance of integrating clinical history, imaging, and pathological findings to achieve accurate diagnosis.

## Introduction

Pleomorphic adenoma (PA) is the most common benign tumor arising from the salivary glands. Approximately 80% of cases occur in the parotid gland, while those originating in the submandibular gland are relatively rare, accounting for about 10% [1,2]. Despite being a benign tumor, PA exhibits a histologically diverse architecture composed of both epithelial and mesenchymal elements and may be associated with cyst formation or calcification [3]. Clinically, it typically presents as a slowly enlarging, painless mass. Although it can occur across a wide age range, it tends to be more common in middle-aged women [1,3].

On imaging, PA often appears as a well-defined, lobulated mass. Although it generally has a fibrous capsule, in some cases, the capsule may be poorly defined or the tumor may protrude beyond the capsule into surrounding tissues [4-6]. As such, distinguishing PA from malignant tumors based solely on clinical presentation or imaging findings can be challenging, and misdiagnosis may occur [1,6]. These misdiagnoses may lead to overtreatment, such as unnecessarily extensive surgery, or undertreatment due to missed malignancy.

Herein, we report a rare case of PA arising from the submandibular gland that was strongly suspected to be malignant based on preoperative imaging and clinical course. We present this case along with a review of the relevant literature, focusing on the diagnostic challenges in differentiating PA from malignant tumors.

## Case presentation

A 66-year-old woman presented to our department with a chief complaint of a mass in the left submandibular region. She had first noticed the mass approximately one month earlier while washing her face. Prior to visiting our clinic, she had consulted an otolaryngologist at another hospital, where a dental origin was suspected, and she was subsequently referred for further evaluation. Her facial appearance was symmetrical, with no signs of swelling or spontaneous pain. On palpation, an elastic, firm mass approximately the size of a thumb tip was detected in the left submandibular area.

**Figure 1:**
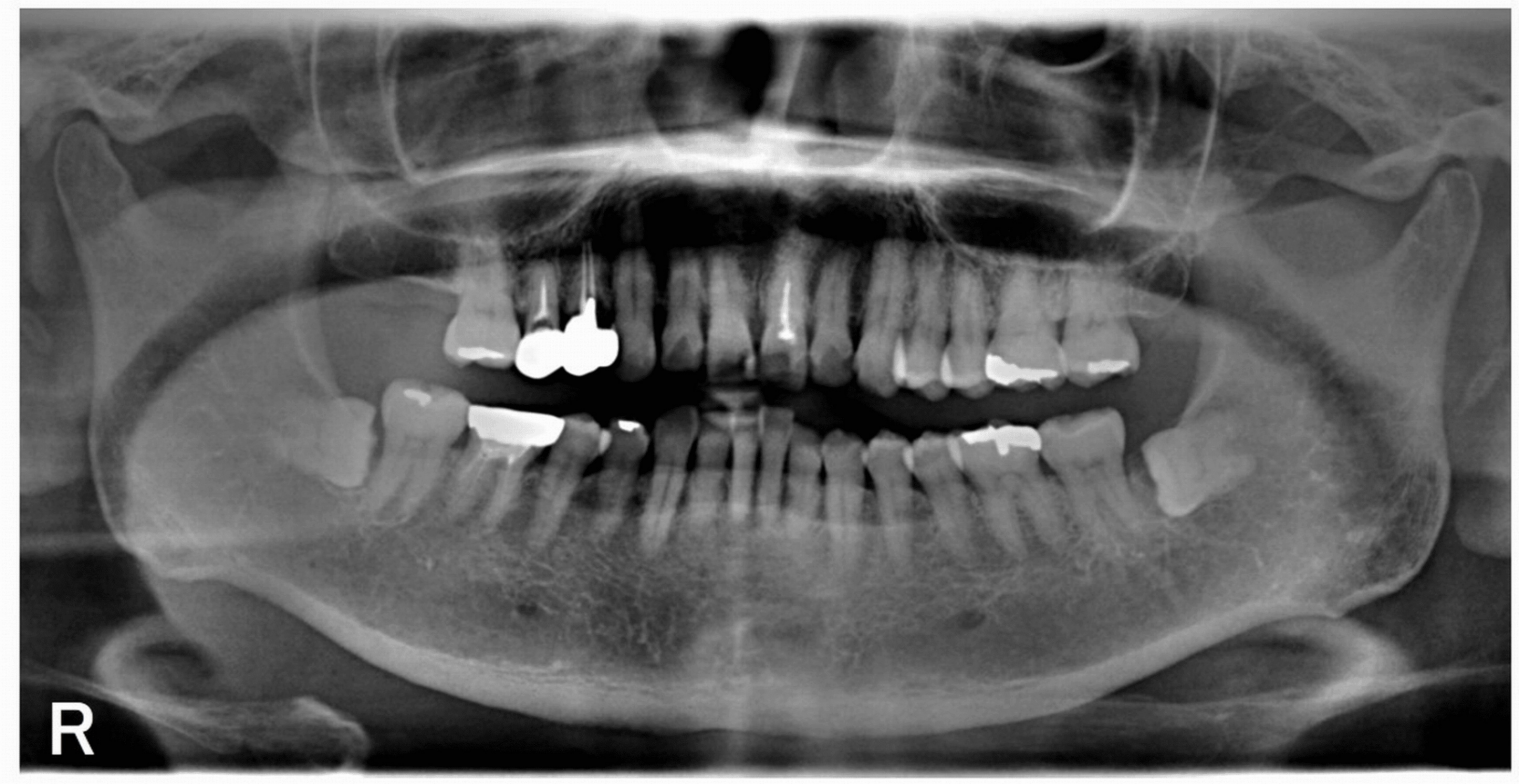
Panoramic radiograph Panoramic radiograph shows no evidence of invasive bone resorption in the left submandibular region.

**Figure 2:**
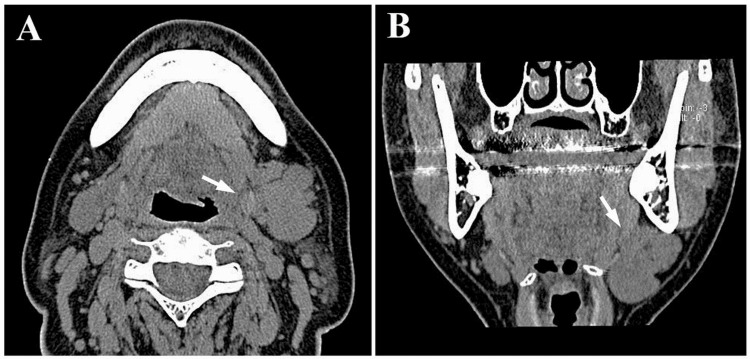
Unenhanced CT images (A: axial; B: coronal) A lobulated mass with irregular margins is observed in the left submandibular gland. The inner border of the lesion is partially indistinct (white arrows), and the internal density is relatively homogeneous. CT: Computed tomography

**Figure 3:**
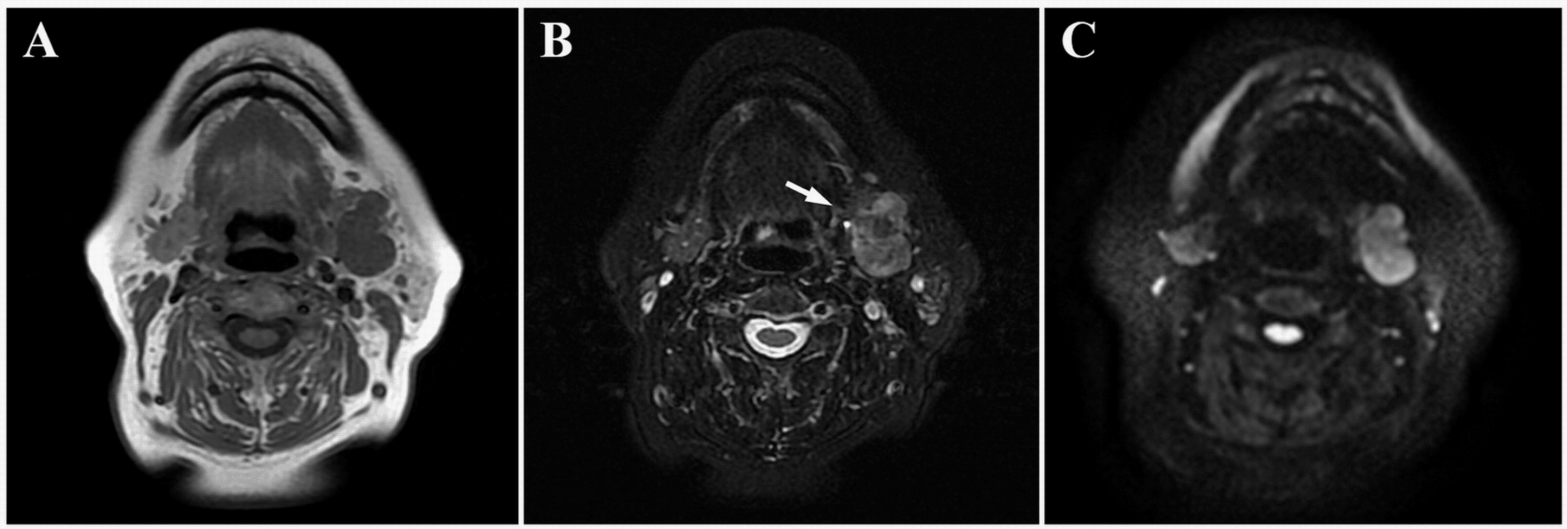
MRI images (A: T1-weighted; B: fat-suppressed T2-weighted; C: DWI) A mass lesion is observed in the left submandibular gland region. The lesion shows relatively homogeneous signal intensity on the T1-weighted image (A). On the fat-suppressed T2-weighted image (B), the lesion presents a lobulated shape with mixed low to high signal intensities and partially indistinct capsular structures (white arrow). The DWI (C) demonstrates heterogeneous high signal intensity. MRI: Magnetic resonance imaging; DWI: Diffusion-weighted imaging

**Figure 4:**
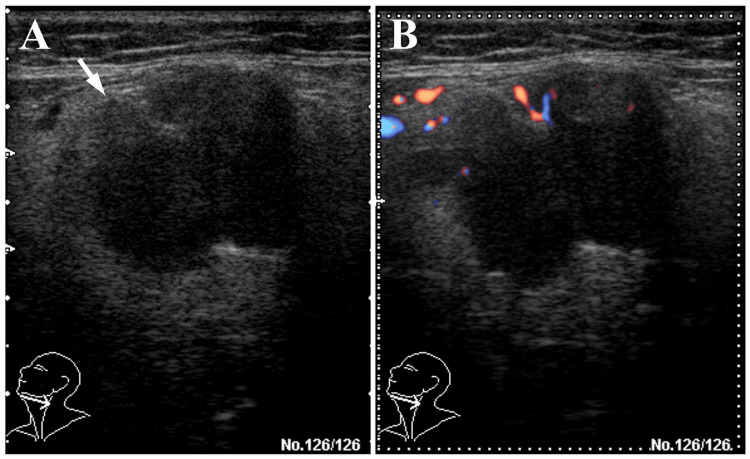
Ultrasonographic images of the left submandibular gland (A: grayscale; B: color Doppler) A lobulated, partially ill-defined (white arrow in A), heterogeneously hypoechoic mass is observed in the left submandibular gland region. Color Doppler imaging demonstrates abundant vascularity within and around the lesion (B).

**Figure 5:**
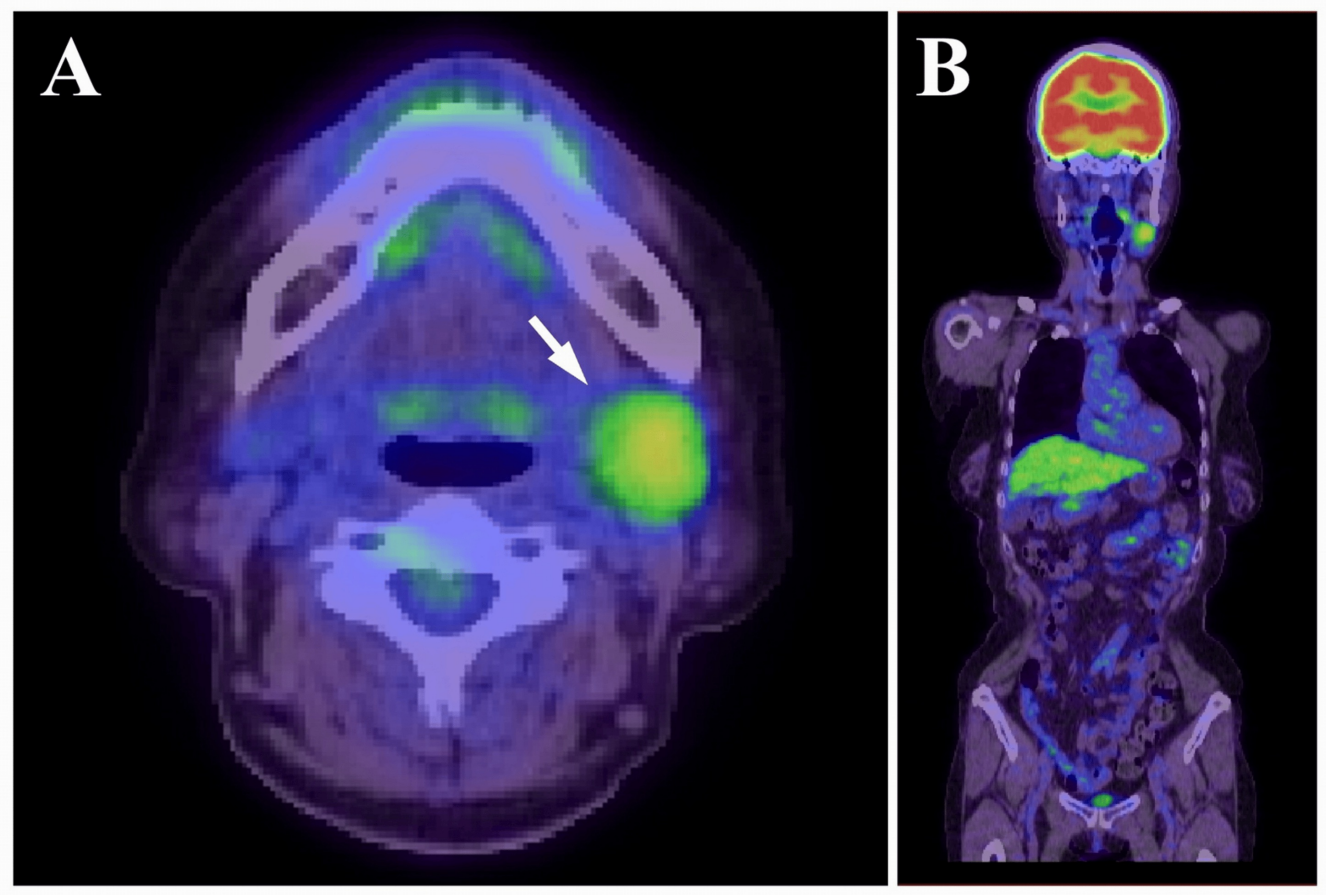
PET images (A: axial, B: coronal whole-body) Abnormal FDG uptake is observed in the left submandibular gland (white arrow in A), indicating a metabolically active lesion. No evidence of distant metastasis, including to the lungs, is seen. PET: Positron emission tomography; FDG: Fluorodeoxyglucose

The panoramic radiograph showed no abnormalities such as invasive bone resorption in the left submandibular region (Figure 1). Unenhanced computed tomography (CT) revealed a lobulated mass with irregular margins in the left submandibular gland region (Figures 2A-2B). The lesion exhibited a partially indistinct inner border, and its internal density was relatively homogeneous. Magnetic resonance imaging (MRI) showed a mass lesion in the left submandibular gland region with relatively homogeneous internal signal intensity on T1-weighted images (Figure 3A). On fat-suppressed T2-weighted images, the lesion exhibited a mixture of low to high signal intensities, had a lobulated shape, and showed partially indistinct capsular structures (Figure 3B). There was no clear evidence of invasion into the surrounding muscles, including the masseter, medial pterygoid, and/or mylohyoid muscles. Additionally, no abnormalities were observed in the cervical lymph nodes on MRI. Diffusion-weighted imaging (DWI) demonstrated heterogeneous high signal intensity (Figure 3C). Ultrasonography (US) revealed a lobulated, heterogeneously hypoechoic mass measuring approximately 21 mm × 24 mm in the left submandibular gland region. The lesion was partially ill-defined and exhibited abundant vascularity both at the periphery and internally (Figures 4A-4B). Positron emission tomography (PET) revealed abnormal fluorodeoxyglucose (FDG) accumulation in the left submandibular gland, approximately 20 mm × 24 mm in size, with a standardized uptake value (SUVmax) of 3.70 (Figure 5A). No distant metastasis, including to the lungs, was identified (Figure 5B).

**Figure 6:**
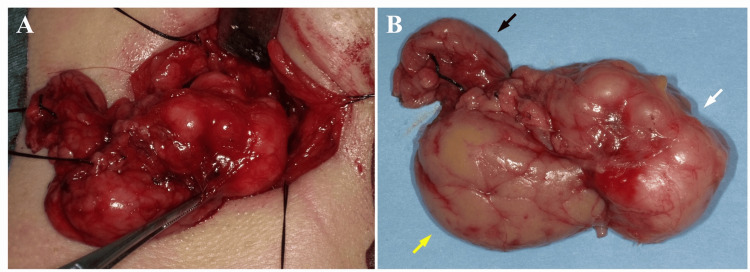
Intraoperative and gross specimen images of the left submandibular gland region (A: surgical field; B: excised lesion) The tumor is identified in the submandibular gland region during surgery (A). The excised lesion shows a lobulated contour (B; white arrow: tumor, yellow arrow: submandibular gland, black arrow: portion of the sublingual gland).

Based on these imaging findings, particularly the irregular margins, partial capsular deficiency, heterogeneous internal structure, and relatively elevated SUVmax, malignancies such as adenoid cystic carcinoma, mucoepidermoid carcinoma, or carcinoma ex pleomorphic adenoma (CXPA) were suspected. Surgical resection was performed under general anesthesia. The tumor was contiguous with the submandibular gland and was carefully dissected to avoid damaging the capsule (Figure 6A). It was excised en bloc together with the entire submandibular gland and a portion of the sublingual gland (Figure 6B). The postoperative course was favorable, with no recurrence observed during 8.5 years of follow-up.

**Figure 7:**
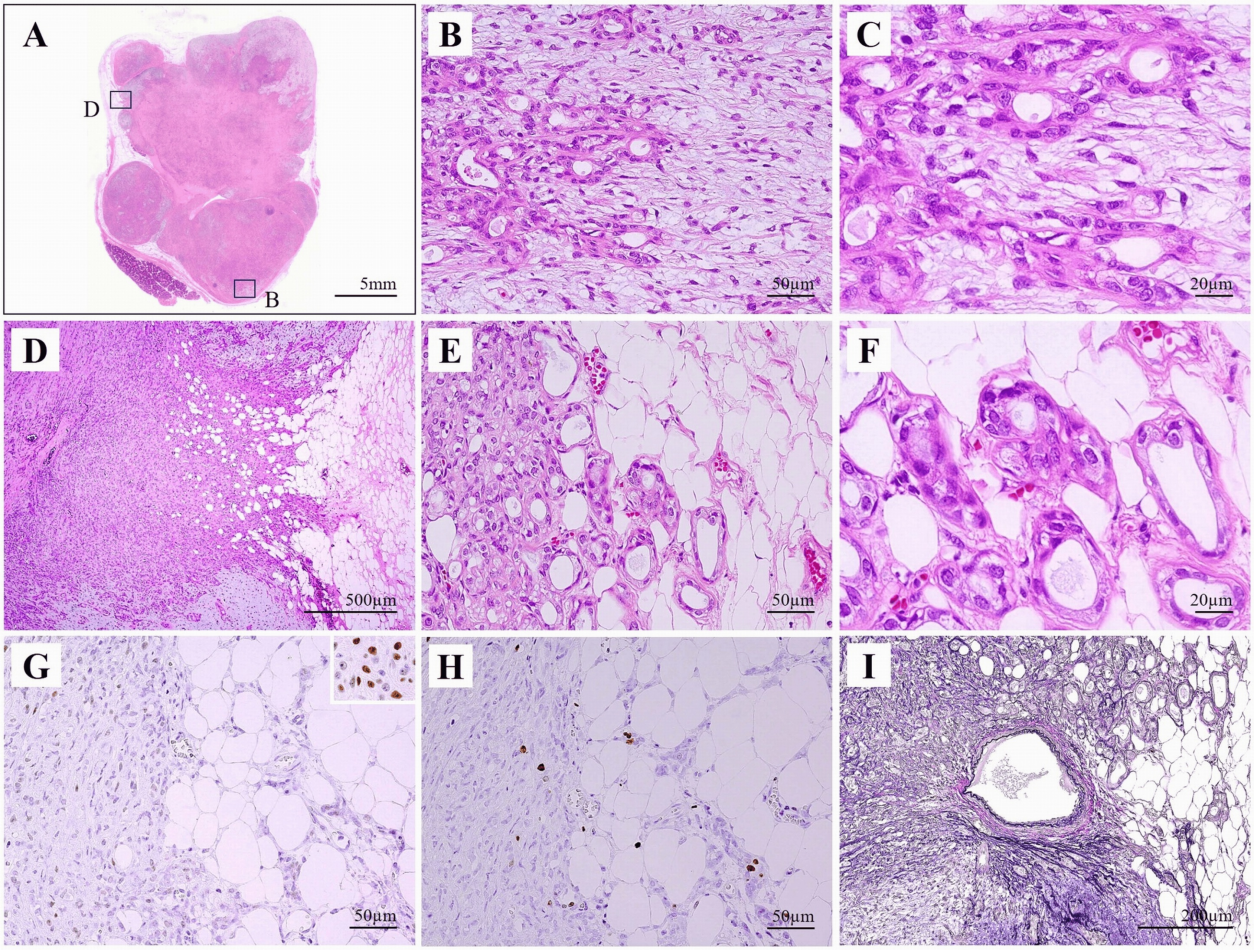
Histopathological and immunohistochemical features of the submandibular gland tumor (A) Multinodular tumor composed predominantly of solid proliferation. (B) Ductal epithelial-like cells and spindle-shaped myoepithelial-like cells form glandular structures with myxoid stromal changes. (C) Some gland-forming tumor cells exhibit bizarre nuclear morphology without marked chromatin hyperplasia. (H&E staining; A: loupe view, B: low magnification, C: high magnification). (D, E) Nests of tumor cells infiltrate the surrounding adipose tissue to a depth of approximately 0.5 mm in areas where the capsule is indistinct. (F) Nests of tumor cells exhibit bizarre nuclei. (H&E staining; D: low magnification, E: intermediate magnification, F: high magnification). (G) Immunohistochemical staining for p53 shows no or weak expression in the tumor cells. The inset shows the positive control. (H) Ki-67 immunostaining demonstrates a low labeling index. (I) Elastica van Gieson staining reveals no evidence of vascular invasion by tumor cells. H&E: Hematoxylin and eosin

Histopathological examination revealed that the tumor was composed of multinodular lesions, predominantly solid proliferation of ductal epithelial-like cells and spindle-shaped myoepithelial-like cells forming glandular structures (Figures 7A-7B). Myxoid changes were also observed within the lesion (Figure 7B). Some of the gland-forming tumor cells exhibited bizarre nuclei without marked chromatin hyperplasia (Figure 7C). In areas where the capsule was indistinct, nests of cells with bizarre nuclei appeared to infiltrate the surrounding adipose tissue to a depth of approximately 0.5 mm (Figures 7D-7F). Immunohistochemical staining was performed using p53 and Ki-67 (Figures 7G-7H), which are commonly employed markers to evaluate malignant transformation in PA [7,8]. Although the glandular epithelium appeared to be non-encapsulated and exhibited an infiltrative growth pattern, there was no overexpression of p53 (Figure 7G), and the Ki-67 labeling index was low, with a maximum of 2.8% (Figure 7H). Furthermore, Elastica van Gieson (EVG) staining showed no evidence of vascular invasion by tumor cells (Figure 7I). Taken together, these findings suggested that true malignant invasion was not present. Although the tumor cells were observed in close proximity to the surgical margin, the margin itself was negative. Based on these findings, the lesion was ultimately diagnosed as a PA of the submandibular gland.

## Discussion

PA is classified as a benign epithelial tumor of the salivary glands as per the 2022 World Health Organization (WHO) classification, accounting for approximately 50-70% of all salivary gland tumors and representing the most common benign neoplasm in this group [1,9]. It predominantly originates in the parotid gland, whereas occurrence in the submandibular gland is relatively uncommon [9]. Clinically, submandibular PA typically manifests as a painless, slowly enlarging, elastic, firm nodular mass in the submandibular region, with cervical lymphadenopathy and neurological deficits rarely observed [1,2].

Radiologic evaluation plays a central role in the diagnostic process of PA. Various imaging modalities, including CT, MRI, and US, are commonly employed [2,3,6,10]. Among them, MRI is particularly valuable for qualitative assessment. Typical MRI findings of PA include low to intermediate signal intensity on T1-weighted images and high signal intensity surrounded by a low-signal capsule on T2-weighted images [3,10]. Contrast-enhanced CT often demonstrates heterogeneous internal enhancement [10]. Nevertheless, certain cases of PA exhibit radiologic features that overlap with those of malignant tumors, making it difficult to achieve a definitive diagnosis based on imaging alone [11]. In the present case, CT, MRI, and US revealed a lobulated mass with irregular margins, partial capsular deficiency, and indistinct borders. The internal signal and echogenicity were heterogeneous. Additionally, PET imaging demonstrated an SUVmax of 3.70, which was relatively elevated. Although increased FDG uptake can be observed in some cases of PA [12], SUVmax values exceeding 3.0 warrant careful clinical consideration [13]. Based on these findings, malignancies such as adenoid cystic carcinoma, mucoepidermoid carcinoma, or CXPA were strongly suspected preoperatively. Moreover, since approximately 50% of submandibular gland tumors are reported to be malignant [2,14], even subtle radiological suspicion should prompt clinicians to consider the possibility of malignancy.

Although pathological examination was not performed preoperatively in this case due to strong suspicion of malignancy based on imaging, procedures such as fine-needle aspiration cytology (FNAC), core needle biopsy, and incisional biopsy are generally useful in the diagnostic workup of salivary gland tumors [15,16]. However, in the present case, invasive diagnostic procedures were deliberately avoided due to the risk of tumor dissemination or capsular rupture. Incisional biopsy allows for the collection of a larger tissue volume than FNAC or core biopsy, potentially enabling a more accurate diagnosis. However, this approach is more invasive and may pose risks such as tumor cell seeding beyond the capsule if incomplete excision occurs. In recent years, endoscopic ultrasound-guided fine needle aspiration (EUS-FNA) has been recommended. This method offers the advantage of being minimally invasive, technically straightforward, and associated with a lower risk of tumor dissemination, making it highly useful for determining treatment strategies [2,17].

Histologically, PA exhibits marked morphological heterogeneity with variable features, even within a single tumor. PA consists of epithelial components, comprising ductal epithelial cells and neoplastic myoepithelial cells, and mesenchymal-like stromal components such as myxoid and chondroid areas. The ratio of these components can vary widely from case to case [3]. Typically, PA is encapsulated by a fibrous capsule, and the presence and continuity of this capsule, along with the absence of extracapsular invasion, are critical factors in diagnosis and surgical planning. However, incomplete capsule formation, pseudopodia (protrusions of the tumor beyond the capsule), and satellite nodules have been reported in some cases [4,5]. These features increase the risk of tumor cell spillage and have been identified as significant risk factors for recurrence [1,18]. In the present case, histological examination revealed regions where the capsule was indistinct, consistent with the radiological findings. In these areas, bizarre-appearing tumor cells infiltrating the surrounding stroma were observed, mimicking malignant invasion. Immunohistochemical analysis was therefore performed, which ruled out malignant transformation of the PA. Based on these findings, although the tumor showed morphologically suspicious features suggestive of malignancy, it was considered benign from an immunophenotypic perspective and ultimately diagnosed as PA.

Surgical excision remains the primary treatment for PA [1-3]. When capsular penetration or extracapsular extension is present, or when surrounding tissues are incompletely excised, recurrence may occur. Therefore, resection including a margin of normal tissue is recommended [1,3,16]. Intraoperative rupture of the capsule significantly increases the risk of recurrence, which has been reported to be approximately 5% [19,20]. Moreover, long-standing PA may undergo malignant transformation into CXPA, highlighting the importance of complete excision during the initial surgery and the need for long-term follow-up [3,19]. In rare instances, PA may invade blood vessels, and metastasis to lymph nodes, bone, skin, liver, and lung has been reported [20]. In the present case, although multimodal imaging raised strong suspicion of malignancy preoperatively, there was no evidence of cervical lymphadenopathy or distant metastasis. Based on these findings, potential overtreatment, such as elective neck dissection or adjuvant therapy, was deliberately avoided. The tumor was resected en bloc under general anesthesia, with careful preservation of the capsule, together with the entire submandibular gland and a portion of the sublingual gland. The postoperative course was favorable, and no recurrence was observed during long-term follow-up of 8.5 years.

## Conclusions

This case underscores the diagnostic limitations of relying solely on imaging modalities to differentiate PA from malignant salivary gland tumors, especially when the tumor originates in the submandibular gland and presents atypical features such as incomplete encapsulation or local infiltration, which may also be seen in PA. In this case, multiple imaging modalities, including CT, MRI, US, and PET, demonstrated findings such as lobulated contours, irregular margins, heterogeneous internal characteristics, and indistinct or partially absent capsule structures, which collectively raised strong suspicion of malignancy. The final diagnosis of PA was established through comprehensive histopathological and immunohistochemical analysis, despite morphologic features that mimicked malignant invasion. This case highlights the importance of integrating clinical findings, multimodal imaging, and detailed pathological evaluation in the diagnostic workup of salivary gland tumors. It provides valuable insights for improving diagnostic accuracy in future clinical practice.

## References

[1] İnan S, Aydın E, Babakurban ST, Akçay EY (2016). Recurrent pleomorphic adenoma of the submandibular gland. Turk Arch Otorhinolaryngol.

[2] Khanal P (2019). Pleomorphic adenoma of the submandibular gland: a case report. JNMA J Nepal Med Assoc.

[3] Lingam RK, Daghir AA, Nigar E, Abbas SA, Kumar M (2011). Pleomorphic adenoma (benign mixed tumour) of the salivary glands: its diverse clinical, radiological, and histopathological presentation. Br J Oral Maxillofac Surg.

[4] Mantsopoulos K, Goncalves M, Koch M, Iro H, Agaimy A (2018). Submandibular gland pleomorphic adenoma: histopathological capsular characteristics and correlation with the surgical outcome. Ann Diagn Pathol.

[5] Mantsopoulos K, Thimsen V, Gostian AO (2022). Histopathology of parotid pleomorphic adenomas: a “pleomorphic approach” to a demanding lesion. Laryngoscope.

[6] Shen Q, Xiang C, Han Y, Li Y, Huang K (2025). The value of multi-phase CT based intratumor and peritumoral radiomics models for evaluating capsular characteristics of parotid pleomorphic adenoma. Front Med (Lausanne).

[7] Di Palma S, Skálová A, Vanìèek T, Simpson RH, Stárek I, Leivo I (2005). Non-invasive (intracapsular) carcinoma ex pleomorphic adenoma: recognition of focal carcinoma by HER-2/neu and MIB1 immunohistochemistry. Histopathology.

[8] Ihrler S, Weiler C, Hirschmann A (2007). Intraductal carcinoma is the precursor of carcinoma ex pleomorphic adenoma and is often associated with dysfunctional p53. Histopathology.

[9] (2024). Pleomorphic adenoma. Head and Neck Tumours. WHO Classification of Tumours, 5th Edition, Volume 9.

[10] Kakimoto N, Gamoh S, Tamaki J, Kishino M, Murakami S, Furukawa S (2009). CT and MR images of pleomorphic adenoma in major and minor salivary glands. Eur J Radiol.

[11] Joe VQ, Westesson PL (1994). Tumors of the parotid gland: MR imaging characteristics of various histologic types. AJR Am J Roentgenol.

[12] Uchida Y, Minoshima S, Kawata T (2005). Diagnostic value of FDG PET and salivary gland scintigraphy for parotid tumors. Clin Nucl Med.

[13] Toya R, Saito T, Matsuyama T (2020). Diagnostic value of FDG-PET/CT for the identification of extranodal extension in patients with head and neck squamous cell carcinoma. Anticancer Res.

[14] Shigeishi H, Ohta K, Okui G (2015). Clinicopathological analysis of salivary gland carcinomas and literature review. Mol Clin Oncol.

[15] Okura M, Hiranuma T, Shirasuna K, Matsuya T (1996). Pleomorphic adenoma of the sublingual gland: report of a case. J Oral Maxillofac Surg.

[16] Sun G, Yang X, Tang E, Wen J, Lu M, Hu Q (2010). The treatment of sublingual gland tumours. Int J Oral Maxillofac Surg.

[17] Jan I, Chung P, Weng M (2008). Analysis of fine-needle aspiration cytology of the salivary gland. J Formos Med Assoc.

[18] Park GC, Cho KJ, Kang J, Roh JL, Choi SH, Kim SY, Nam SY (2012). Relationship between histopathology of pleomorphic adenoma in the parotid gland and recurrence after superficial parotidectomy. J Surg Oncol.

[19] Valstar MH, de Ridder M, van den Broek EC (2017). Salivary gland pleomorphic adenoma in the Netherlands: a nationwide observational study of primary tumor incidence, malignant transformation, recurrence, and risk factors for recurrence. Oral Oncol.

[20] Marioni G, Marino F, Stramare R, Marchese-Ragona R, Staffieri A (2003). Benign metastasizing pleomorphic adenoma of the parotid gland: a clinicopathologic puzzle. Head Neck.

